# Regulation of locomotor pointing across the lifespan: Investigating age-related influences on perceptual-motor coupling

**DOI:** 10.1371/journal.pone.0200244

**Published:** 2018-07-19

**Authors:** Steven van Andel, Michael H. Cole, Gert-Jan Pepping

**Affiliations:** School of Exercise Science, Australian Catholic University, Banyo, QLD, Australia; University of Illinois at Urbana-Champaign, UNITED STATES

## Abstract

**Introduction:**

The regulation of one’s step length by placing one’s foot at a specific position within gait, otherwise known as ‘locomotor pointing’, is well understood in walking and running gait. The current study was the first to broaden this understanding to a larger cohort and to describe the influence of age on the regulation of locomotor pointing when walking up to and stepping onto a curb-like platform.

**Methods:**

Younger (n = 17, mean age: 25.35 years, range: 19–33) and older adults (n = 105, mean age: 71.49 years, range: 61–86) participated in a walking experiment, requiring them to approach and step onto a curb-like platform. Linear mixed effects modeling was used to study the main outcome variables: onset of regulation, the regulation strategy and the strength of perceptual-motor coupling.

**Results:**

Results showed that with older age, participants showed less variability in foot placement during their approach and seemed to prefer to shorten their steps. Furthermore, the strength of the perceptual-motor relationship was found to be related to age; regulation of step length of both younger and older participants was based on a participant’s current foot position. The strength of this relationship increased as participants got closer to the curb and was stronger with increasing age. Furthermore, younger adults on average lengthened their steps as they got closer to the curb, whereas older adults showed significantly less lengthening compared to their younger counterparts. No age-related differences were found in terms of onset of regulation.

**Discussion:**

The results suggest that the strength of the perceptual-motor relationship in gait is related to age. It is argued that this age-related increase in the strength of perceptual-motor coupling is required to cope with increasing demands linked to the age-related declines of action capabilities. The implications of the findings are discussed in the context of increased falls risks and deficits in perceptual-motor functioning.

## Introduction

In everyday motor control, humans, young and old, show a great capacity to regulate and perform successful actions. For instance, consider a long jumper who is running at near maximal speed, but is still able to guide their foot to the take-off board with amazing accuracy. Research has shown that this type of locomotor pointing is made possible through an intimate coupling of perception and action [1–6]. The regulation required for such a task is not limited to athlete populations, but rather, is also used by members of the general population on a daily basis when performing locomotor pointing tasks, such as approaching and stepping onto a curb [7]. Whilst these studies would suggest that locomotor pointing is a skill that can be comfortably performed by all humans, research concerning age-related changes in locomotor pointing in otherwise healthy populations is limited. The current study addressed this limitation and investigated the effect of age on perceptual-motor coupling in healthy older adults.

Whilst investigating locomotor pointing in the approach of a long jump, Lee and colleagues [2] analyzed the variability in foot placements leading up to the jump and identified two separate phases in the regulation of foot placements. In the first phase, the variability in foot placements accumulated as the athlete attempted to produce a stereotypical stride pattern to optimize acceleration. In the second, ‘zeroing-in’ or regulation-phase, the athletes were shown to reduce the accumulated variability in foot placement to initiate the jump from as close to the take-off board as possible [2]. Later research assessed when participants initiate this regulation-phase and showed that this did not contain a fixed number of steps in each trial [1]. Rather, findings showed that the onset of regulation for individual trials was related to the total amount of adjustment required. That is, if an athlete required large adjustments to end up with minimum error at the take-off board, he or she would start regulation earlier. Furthermore, research showed that the adjustments shown during the run-up were achieved through a tight relationship between perception and action throughout the final steps in a run-up. That is, results showed that the produced adjustments in a single step were linearly related to the adjustments required, based upon the previous foot-position of the athlete. In such an assessment of the relationship between the produced adjustment and a person’s position, a strong linear relationship can only be formed when a person bases their adjustment of step length on their perception of their location. In accordance with other research [1–6], in the current study this is interpreted as evidence for *perceptual-motor coupling*. A previous study on locomotor pointing in the long jump run up that similarly interpreted the relationship between produced and required foot-placement adjustments as perceptual-motor coupling reported that this relationship was established about four steps before the jump and that it became increasingly stronger (evidenced by an increasing steepness of the regression line) as the athletes drew closer to the target [1].

In subsequent research, it was shown that people exhibit the same perceptual-motor coupling strategy as shown in the long-jump in other locomotor pointing tasks, such as walking to a target [7–12]. In these studies, the relationship between the gait adjustments required and gait adjustments produced was measured for each step in the approach to a target as the degree to which deviations from the average step length were related to the adjustment required. The adjustment required was measured as the difference between the location of the foot during the approach (e.g. the fifth last step before reaching the target) and the mean location of that foot for that step (i.e. the mean location of the foot during the fifth last step before reaching the target). Similarly, adjustment produced was measured as the difference between a certain step’s length and the average step length [1,5,9,12]. A recent study described the strength of this relationship between adjustment required and adjustment produced as representing the ‘strength of perceptual-motor coupling’ [7]. In this interpretation, a stronger coupling recorded for a specific step would indicate that more of the required adjustment is being made in that specific step. For instance, a 100% coupling would indicate that if, at a certain step, a person is 5cm behind on his/her average approach; they will adjust their next step to be 5cm longer than their average step. Data over multiple walks can be analyzed using regression analyses for each step, with a greater steepness (beta values close to 1, indicating close to 100% correction in one step) indicating stronger perceptual-motor coupling. In a small number of studies, the influence of changing task constraints on locomotor pointing behavior has been investigated [8,12] and it was shown that tasks with higher spatiotemporal demands (e.g. smaller targets or higher movement speeds) led to stronger perceptual-motor coupling as indicated by higher beta values in the regression analysis. For instance, high spatiotemporal demands in a walking task have been shown to lead to beta values of around 0.71 in a single step, compared to beta values of around 0.39 for walking tasks with lower demands [8].

Research into locomotor pointing, to date, has shown that even though aspects of onset and strength of perceptual-motor regulation might differ between locomotor pointing tasks, all tasks seem to share common principles. That is, similarities in the regulation of foot placement exist between tasks performed in high-performance settings (e.g. long jump), as well as in everyday activities, such as walking to and stepping onto targets [8] and approaching and stepping onto a curb [7]. This is helpful in broadening the understanding of perceptual-motor coupling in locomotor settings. Further, it provides a vehicle for assessing perceptual-motor coupling in cohorts of participants that cannot cope with the high demands of athletic locomotor tasks, such as a long jumping approach. In the current investigation we used a walking approach to stepping up a curb to study locomotor pointing in a cohort of otherwise healthy younger and older adults. This task is different from traditional locomotor pointing research in that the act of stepping onto a curb has no clearly defined target. In such situations where movement is less constrained by a target, one can expect later initiation of regulation and a less strong perceptual-motor coupling [8].

The physical and functional decline of an individual’s capabilities with age has been well documented [13–15]. These declines in the motor components of the perceptual-motor system mean that older adults need to cope with a changing relationship between perceptual and motor function. The current study aimed to describe how regulation of locomotor pointing changes with this changing relationship between perceptual and motor function. This aim was achieved by investigating: 1. the onset of regulation; 2. the strength of perceptual-motor regulation; and 3. how people adjust their steps (i.e. by lengthening or shortening their steps) in a large group of older adults and a group of younger controls. Cornus and colleagues [8] found that, when higher task demands were experimentally implemented, the strength of perceptual-motor regulation increased. It is hypothesized that the age-related declines to their motor systems experienced by older adults lead to comparatively higher task demands, and similar to the effects described by Cornus [8], this would lead to stronger perceptual-motor regulation.

## Methods

### Participants

Two groups of participants volunteered to be part of the experiment. The first group consisted of 17 younger participants (mean age: 25.35 years, SD: 3.76 years, range: 19–33, 10 males, 7 females). The second group composed 105 older adults (mean age: 71.49 years, SD: 5.60 years, range: 61–86 years, 29 males, 76 females) from a local community of healthy older adults. All participants had normal or corrected to normal vision, were free of leg injury or known balance disorders, and were able to stand and walk without the use of a walking aid for the entire length of the experiment. The younger group was required to be aged between 18 and 40 years (similar to the age ranges used in previous perception-action research [16]), while the older participants were eligible to participate if they were aged over 60 years and had no cognitive deficits (measured as Mini Mental State Examination scores > 23). The protocol of the study was identical for both groups and was approved by the Australian Catholic University's Human Research Ethics Committee (2015-306H) and all participants signed an informed consent form.

### Protocol and materials

**Fig 1:**
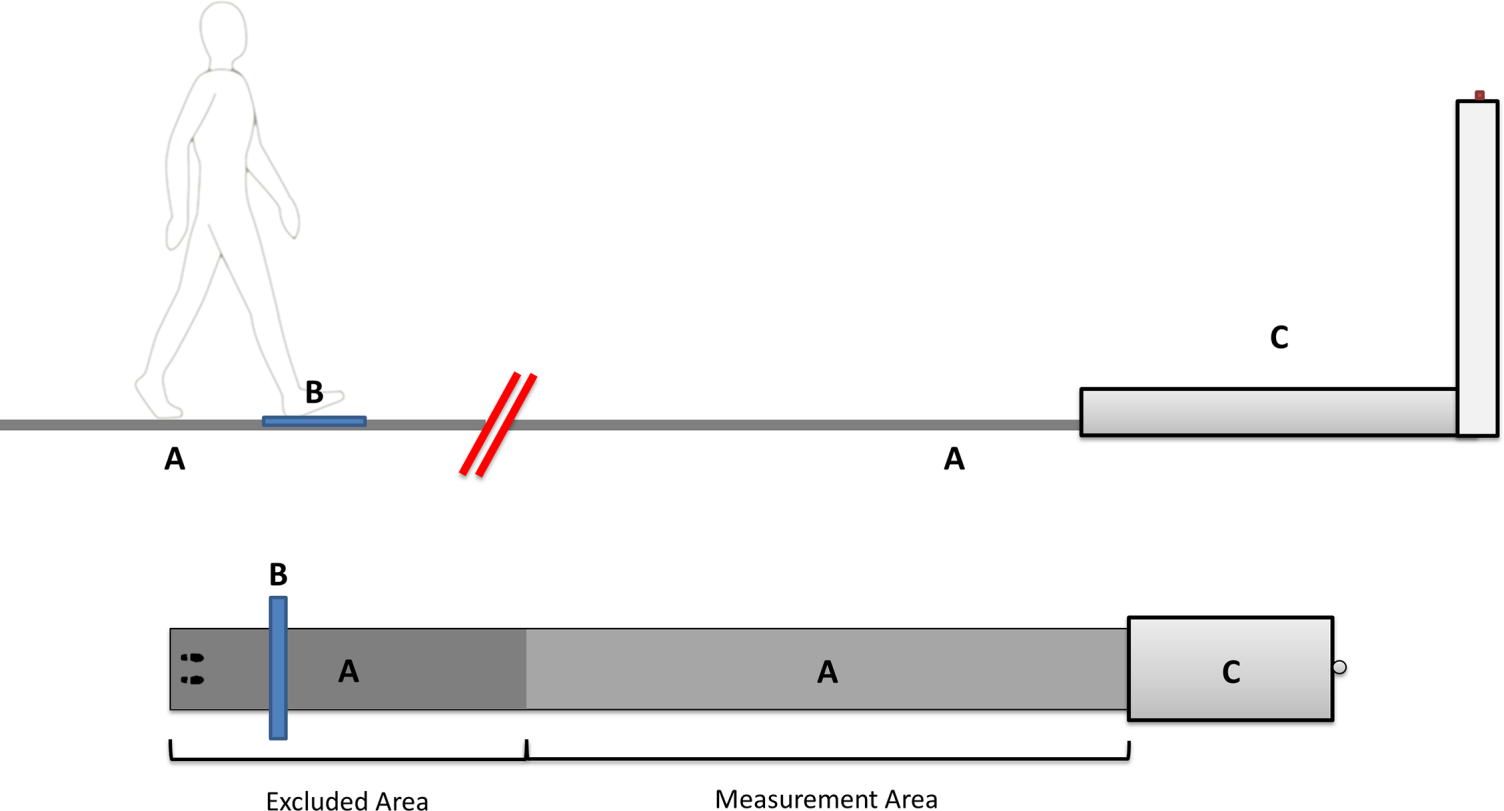
Schematic of the experimental set up. A) the 8.5-meter pressure-sensitive walkway, B) the moveable target, and C) the platform (15 cm high, 100 cm wide and 200 cm long) with the flick button at the end (at 1.35 m high).

Participants completed a series of walking tasks that involved walking along an 8.5-meter pressure-sensitive walkway (GAITRite, CIR Systems Inc.) to a purpose-built platform that they stepped up and onto (Fig 1). The platform measured 15 cm high, 2 meters long and 1 meter wide and was designed to conform to standard curb building regulations in Queensland, Australia. For each trial, participants were instructed to walk the full length of the walkway, step up onto the platform and press a switch that was situated at a height of 1.35 meters at the end of the platform. A measure of average step length was established a-priori, to be used for setting up the main experiment. To record this a-priori measure, participants performed three unconstrained walks over the pressure-sensitive walkway. A fourth walk was added if the experimenter judged one of the initial three walks to be dissimilar to the others in terms of step length (some participants approached the first walk rather cautious, making the average not representative of their natural gait). No further outcomes were derived from these a-priori walks.

To facilitate comparison of the current study with previous research and to prevent the participants from producing the same stereotypical walk each time, they were required to place one of their early steps onto a target on the ground (a blue anti-slip mat, dimensions: 30 cm long and 150 cm wide), which was placed in 10 different positions ranging from 1 to 2.5 times the a-priori measured step lengths from the start of the walkway [7]. In addition, a final condition was added in which no early stepping target was presented and the participants completed the task unconstrained. All 11 conditions were repeated three times in a random order to produce a total of 33 walks per participant.

The inclusion of the early step target prevented the participants from producing the same stereotypical walk each time by influencing the participants’ step length early in their approach. In order to not have this influence any outcome measures, footfalls placed in the first 3.5 meters of the GAITRite were excluded from the analysis. Hence, all the results reported were collected from the final five meters of the walk before stepping onto the curb (Measurement Area in Fig 1)

Information about the placement of each footfall before stepping onto the curb was collected using the GAITRite system and exported to Microsoft Excel. A digital video camera (CASIO, EX-FH100), placed 2.35 m from the edge of the platform, perpendicular to the direction of walking was used to collect information regarding the participants’ step onto the curb. In calculating the participants’ step lengths towards this position on the curb, only the horizontal distance between subsequent steps in the sagittal plane was analyzed. The Kinovea software (version 0.8.15, ©2006–2011 Joan Charmant & Contrib.) was used to analyze the video data and to extract the first foot position in the direction of walking on top of the curb for each walk [7]. An assessment of inter-rater reliability was performed by three independent raters based on the videos for two participants (66 trials). The foot placements derived for these trials were shown to have very high repeatability (ICC = 0.993). Further calculations and the statistical analyses were performed using MATLAB (version R2015a, © 1984–2015 The MathWorks, Inc.).

### Variables

The following variables were extracted from the dataset and used in the statistical analysis.

#### Variability in footfall position (SD-footfall)

The variability in footfall position *(SD-footfall)* was calculated for each footfall, and for each participant to show how variability in positioning changed as participants got closer to the target. As the variability in foot placement is partly dependent on the length of one’s steps (and results could thus be attributed to step length differences, rather than age), a scaled measure of SD-footfall was used in all analyses (SD-footfall/Step Length).

#### Standard step length (SSL)

The participants standard step length *(SSL)* was calculated by averaging the length of the 4 steps starting at least 3.5 meters away from the starting position, for each trial. The standard deviation around this mean was referred to as the ‘SSL-SD’.

#### Onset of regulation (OnsetReg)

The onset of regulation *on a trial-by-trial level* was designed to identify when significant adjustments are made in a person’s step lengths. *OnsetReg* was calculated relative to SSL. If a step in was more than two times SSL-SD different from this SSL for that trial, the step was considered to be an adjusted step [7,8]. The first step to be marked as ‘adjusted’ within a particular trial was marked as OnsetReg for that trial. OnsetReg were analysed in terms of step number as well as distance from the curb. Finally, if at least one of the steps within a trial was considered to be adjusted, the walk itself was marked as *adjusted*. If no steps in the trial were considered to be adjusted, the trial was marked as *unadjusted*.

#### Adjustment per trial (Adjust_total_)

For all steps marked as adjusted, the SSL was subtracted from the length of the adjusted step. As such, when the result was positive, it indicated a *lengthening* step relative to the SSL, while a negative outcome indicated a *shortening* step relative to the SSL. The absolute value of the sum of these deviations of the standard step per trial was computed to assess the total adjustment per trial, or *Adjust*_*total*_. Based on the classification of adjusted steps as being either lengthening or shortening steps, the trials as a whole were categorized for their adjustment strategy; a lengthening strategy (all adjusted steps were longer than the standard steps), a shortening strategy (all adjusted steps were shorter than the standard step length) or a mixed strategy (at least one adjusted step was longer and one was shorter than the standard step).

#### Adjustment required (Adjust_required_,)

*Adjust*_*required*_, was computed for each of the final six foot placements before stepping up and onto the curb-like platform of all adjusted trials, as the difference between the current foot placement and the mean placement for that step, per participant.

#### Adjustment produced (Adjust_produced_)

Similar to *Adjust*_*required*_, the *Adjust*_*produced*_ was computed of the final six steps in adjusted trials as the difference between each step and the average step length at that step per participant.

### Statistics

#### Between group differences in strategy

An ANOVA was used to assess differences in the dominant step length adjustment strategy (number of trials in a particular strategy) between age groups. For this analysis, the cohort of older participants was split into three age groups, based on chronological age and compared with each other and the young group. In grouping the participants, it was aimed to keep sample sizes between groups as equal as possible whilst not sorting people of the same chronological age into different groups.

#### Linear mixed effects modelling

In order to assess the effect of aging on the other variables, a Linear Mixed Effects (LME) Modelling analysis was adopted and these outcomes complemented the above-mentioned analyses. In all LME models, age was included as a continuous variable (in contrast to the between-group analysis above) and the p-value of the coefficients was analyzed to indicate significance of a certain factor and alpha for all models was set to 0.05.

#### Variability of foot placement

The influence of age on footfall variability was studied using an LME model with the step length scaled variability in foot placement (SD-footfall/Step Length) entered as a dependent variable. It was expected that the relationship between age and SD-footfall might change as participants moved closer to the curb-like platform and that this change would be indicative of an adjustment strategy. Therefore, slopes and intervals were allowed to vary per step number (random effect) in the LME model. The formula below describes the model used, where Footfall_number_ indicates the number of the footfall, counting backwards from the first footfall on top of the curb (this being footfall0, the last footfall before stepping up being footfall-1, etc.).

$$\frac{SD-Footfall}{Step\;Length}∼Age+(1+Age|{Footfall}_{number})$$

#### Onset of regulation

A second LME model was set up to study the influence of age on OnsetReg. Intercepts and slopes of the fixed effects were allowed to vary for the different adjustment strategies (random effect). This resulted in the model below.

$$OnsetReg∼{Adjust}_{total}*Age+(1+{Adjust}_{total}*Age|Strategy)$$

#### Step length adjustments

In order to assess the influence of age on step length throughout the walks, the LME model described below was used, in which Footfall_number_ indicated the number of the footfall relative to the curb.

$$SL∼Age+(1+Age|{Footfall}_{number})$$

#### Strength of perceptual-motor coupling

Finally, a LME model was computed to assess the influence of age on the relationship between Adjust_required_ and Adjust_produced_. Age and Adjust_required_ were entered in the model as fixed factors. The intercept and slope of the fixed effects were allowed to vary for each Footfall_number_ (Footfall_number_ being the number of the footfall relative to the first footfall on the curb; footfall0). This resulted in the following LME model:

$${Adjust}_{Produced}∼{Adjust}_{Required}*Age+(1+{Adjust}_{Required}*Age|{Footfall}_{number})$$

## Results

### Between group differences in strategy

Data for eight of the 105 participants in the older participant group (two males, six females, average age: 75 years, SD: 7.13) were lost due to errors related to the video camera (e.g. insufficient memory or battery power). The remaining 97 older participants were subsequently sub-divided into 3 groups based on their chronological age to give; i) a young-old group (61–68 years); ii) a middle-old group (69–73 years); and iii) an old-old group (74–85 years). Although the specific age ranges used to define these groups were not of equal size, they were guided by the need to match these three cohorts as closely as possible for sample size. No data were lost for the younger group (N = 17) and, hence, a total to 114 participants were included in the presented analyses. The descriptive statistics of the participants are reported in Table 1 for the younger participants and the three older participant sub-groups.

**Table 1:** Descriptive statistics (Mean ± SD) for the gait characteristics of participants split into age categories. ‘Standard’ Measures were Derived from the Middle of the Walks, ANOVAs with alpha set to 0.05 were used to Identify Significant Effects of Age Category on all Measures Except ‘Mean Age’.

Measure	Young Age 19–33 yr	Young-Old Age 61–68 yr	Middle-Old Age 69–73 yr	Old-Old Age 74–85 yr
	N = 17	N = 33	N = 35	N = 29
Mean Age (years)	25.35 (3.76)	65.59 (2.19)	70.97 (1.52)	77.96 (3.21)
Standard Step Length (cm) ^b^	75.07 (4.46)	68.64 (8.56)	68.60 (7.54)	67.28 (7.88)
Maximal Comfortable Step Length (cm) ^b^^d^	84.48 (4.21)	75.42 (8.69)	75.10 (8.01)	74.31 (9.14)
Standard Walking Speed (m/s)^a^	1.40 (0.14)	1.31 (0.21)	1.31 (0.14)	1.29 (0.18)
Minimal SD-Footfall (cm) ^b^	11.56 (2.37)	7.00 (2.98)	7.56 (3.23)	7.32 (3.54)
OnsetReg in steps (step number) ^c^	2.70 (0.28)	2.95 (0.28)	2.74 (0.33)	2.95 (0.36)
OnsetReg in distance (cm)^a^	107.37 (19.59)	107.16 (24.94)	95.27 (18.58)	103.56 (18.37

^a^ ANOVA testing revealed no significant differences between groups.

^b^ Bonferroni corrected post-hoc analysis revealed differences between the young group and three older groups, no differences between older groups were found

^c^ Bonferroni corrected post-hoc analysis revealed the Young and Middle-Old groups to be significantly different from the Young-Old and Old-Old groups

^d^ Maximal Comfortable Step Length represents the average over the 11^th^ to the 20^th^ biggest steps, representing a step length that was not on a participant’s absolute upper limit, but rather was a more functional representation of their maximum step.

Table 1 shows that, when assessing the absolute distance to the curb at which participants started to adjust their steps (OnsetReg in distance), no age-related differences were found. However, when assessing differences in terms of step number between age groups (OnsetReg in step number), significant effects indicated that the young and the middle-old group initiated regulation later compared to the young-old and old-old groups.

**Fig 2:**
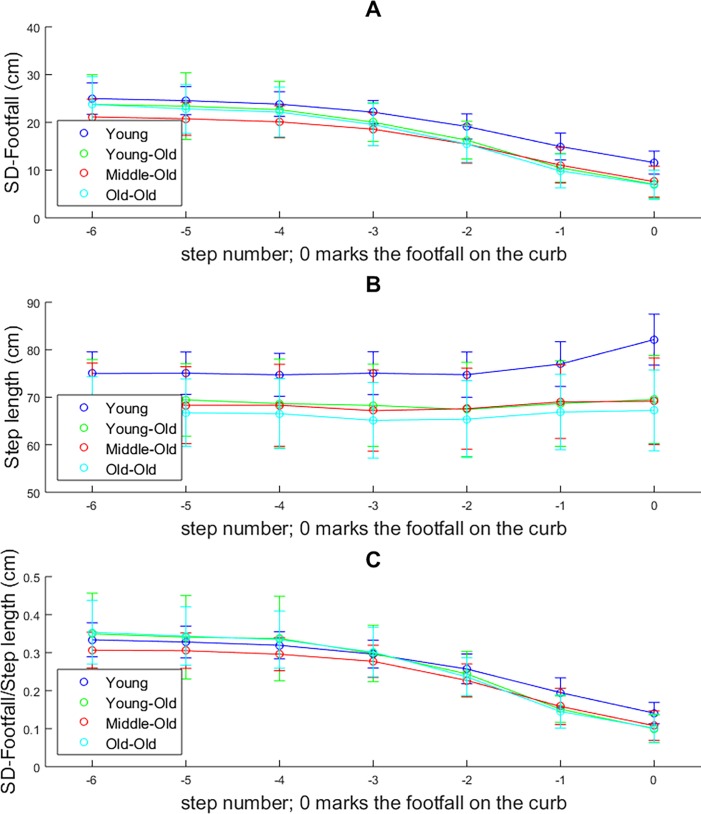
Descriptive analysis of locomotor pointing behavior. Data depicted are split into age categories to make the effects of age more visually discernable; however it should be noted that, in the statistical analysis, age was included as a continuous variable. Panel A shows the standard deviation of footfall position (SD-footfall) as a function of the step number before stepping onto the curb. Panel B shows step length as a function of step number. And panel C shows the SD-footfall scaled by step length for each step number.

Fig 2B shows the average step lengths per group over the approach. It was clear that a lengthening strategy was dominant in the younger cohort. None of older groups showed this lengthening strategy.

**Fig 3:**
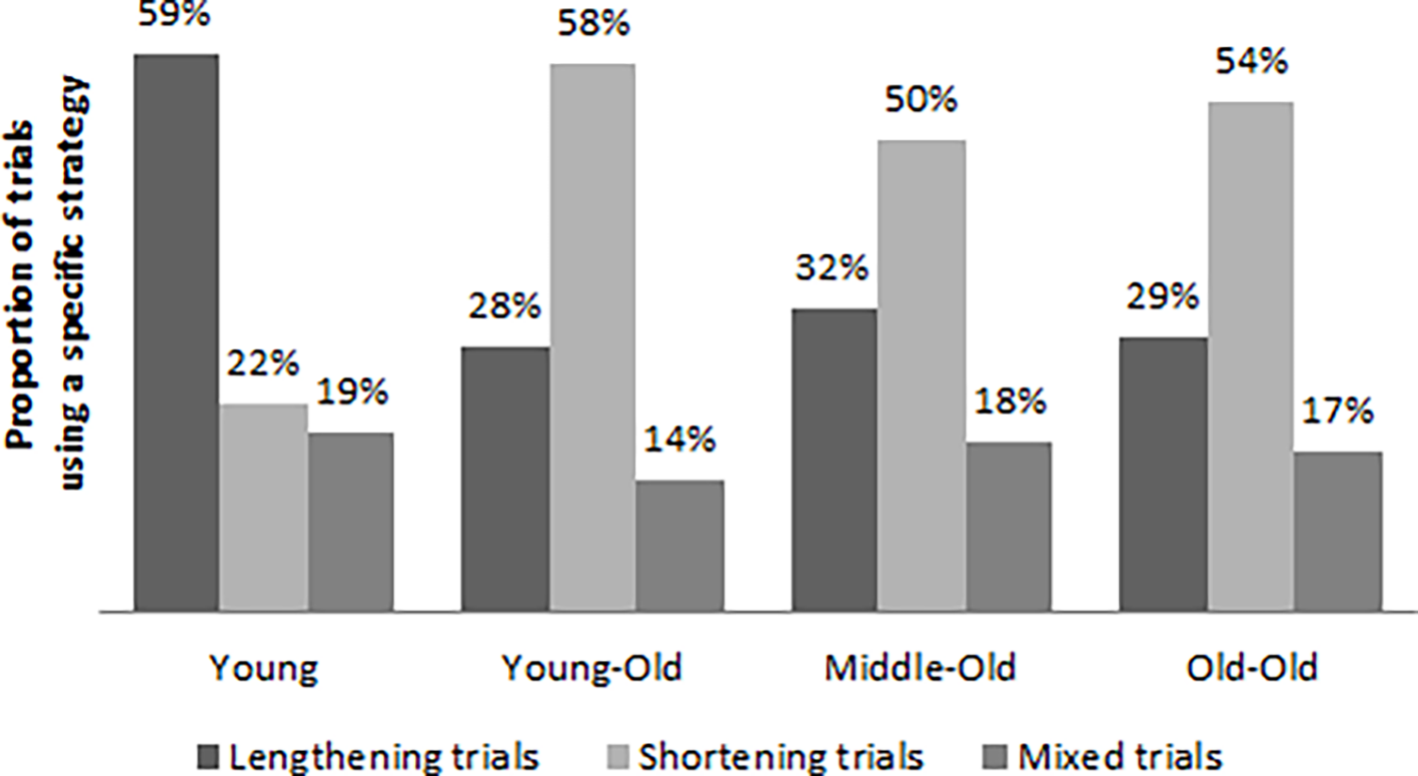
Distribution of lengthening, shortening and mixed trials for the four different age groups.

The distribution of adjustment strategies of step length (lengthening, shortening, mixed) across the different age groups is displayed in Fig 3. Effects of age on the distribution of adjustment strategies were investigated using ANOVA testing. The assumption of normality of the data was assessed using Levene’s test, which identified no violations to the assumption. The analysis returned a significant main effect for group on the number of lengthening (F(3,113) = 8.03, p < 0.001) and shortening (F(3,113) = 7.88, p < 0.001) trials, but not for the number of mixed trials (F(3,113) = 0.82, p = 0.485). Post-hoc analyses with Bonferroni corrections identified that the younger group exhibited a greater number of lengthening trials than the three older groups, while the older groups all adopted a shortening strategy more often than the younger cohort (all p-values < 0.05). No significant differences were identified amongst the older groups.

### Linear mixed effects modelling

#### Variability of foot placement

Results from the LME analysis predicting Footfall-SD corrected for step length are summarized in S1 Table. Overall, the model had a R^2^ of 0.641. The fixed effect for age was significant, indicating that SD-footfall/Step Length decreased with age. Furthermore, a significant random effect for age was seen at footfall0, footfall-1, footfall-4, footfall-5 and footfall-6. For footfall0 and footfall-1, the negative coefficients indicated that advancing age was related to smaller values of SD-footfall. For footfall-4, footfall-5 and footfall-6, the positive coefficients of the random effect of age on SD-footfall were reversed; that is, increased age was related to increased variability.

#### Onset of regulation

S2 Table summarizes the LME model predicting OnsetReg using Adjust_total_ and age as fixed factors and a random factor for adjustment strategy, producing a R^2^ of 0.246. When Adjust_total_ and age were entered in the model together, neither of these fixed factors significantly contributed to the model. Only one random effect reached significance; the OnsetReg occurred later in lengthening trials compared to the other two strategies.

#### Step length adjustments

The LME analysis that sought to predict step length with age entered as a fixed factor and footfall number entered as a random factor is presented in S3 Table and reports the R^2^ for the model as 0.143. The significant fixed effects for age indicated that step length decreased with age. A significant random effect was only present at step0, with the negative coefficient indicating that this step became shorter with increasing age.

#### Strength of perceptual-motor coupling

**Fig 4:**
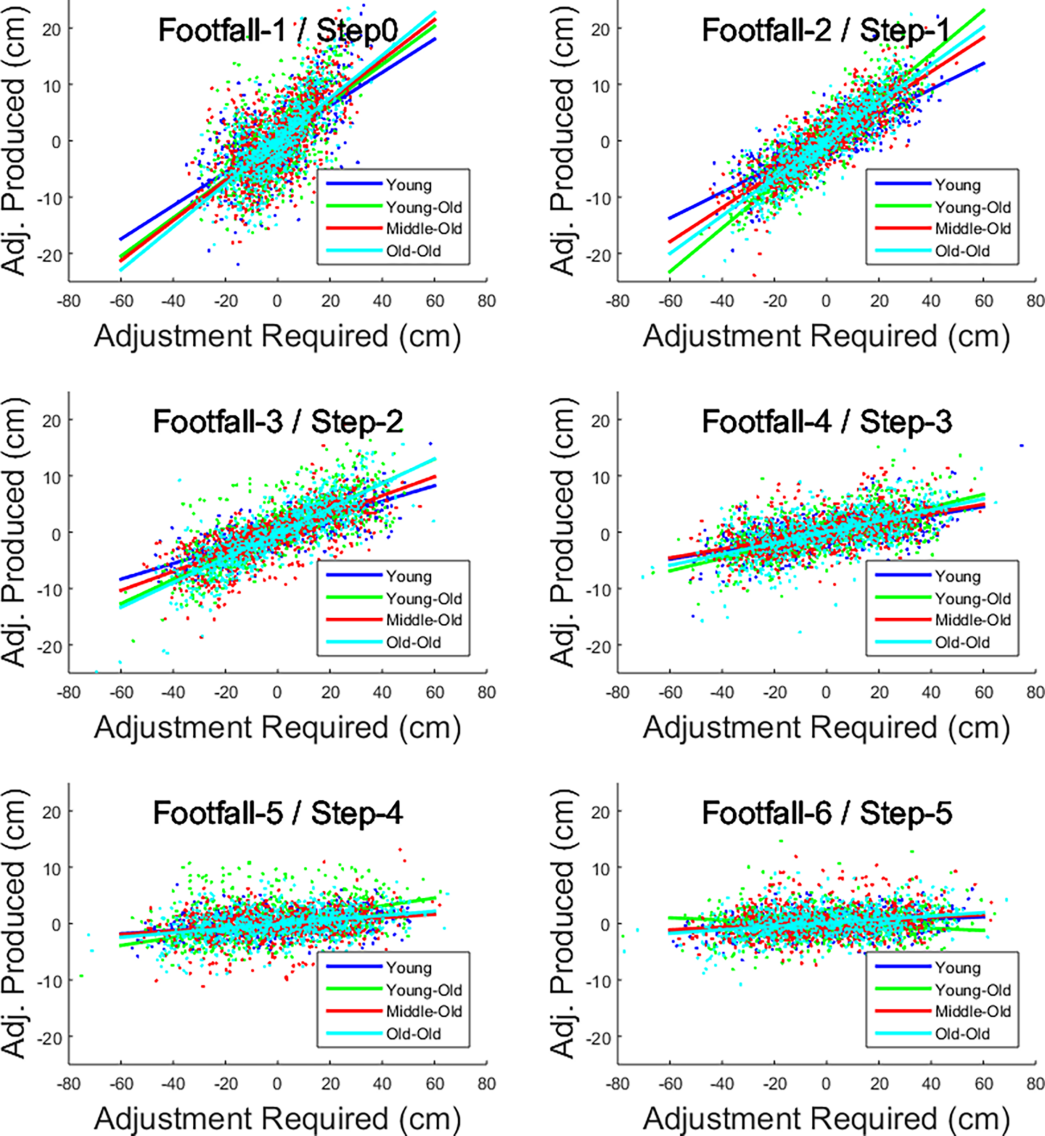
Strength of perceptual-motor coupling. Figure indicating the relationship between Adjust_required_ and Adjust_produced_ for foot placements leading up to the curb. Footfall-1 is the last foot placement before stepping up (step0). Data depicted are split into age categories to make the effects of age more visually discernable, however it should be noted that, in the statistical analysis, age was included as a continuous variable.

The results from the LME analysis relating the relationship between Adjust_required_ and Adjust_produced_ are displayed in the S4 Table and the results are illustrated in Fig 4., with the overall model returning a R^2^ value of 0.442. The significant fixed effect of Adjust_required_ confirmed that the Adjust_required_ was directly related to the Adjust_produced_. The interaction between Adjust_required_ and Age was also significant, indicating that the relationship between Adjust_required_ and Adjust_produced_ became stronger with increasing age. From the analysis of the random effects, the following results became apparent. The effect of Adjust_required_ on Adjust_produced_ was significantly weaker in the early steps (footfall-5 and footfall-6), as indicated by the negative beta values. However, this effect strengthened in the latter steps (footfall-1 and footfall-2), as indicated by the positive beta values returned for the random effect. Furthermore, the interaction between Adjust_required_ and Age showed a similar trend, with negative coefficients in the early steps (footfall-5 and footfall-6) and positive coefficients in the latter steps (footfall-1 and footfall-2). These findings indicated that the effect of age on the relationship between Adjust_required_ and Adjust_produced_ became stronger as the participants drew closer to the step up. Finally, the negative main effect for age at footfall-1 and footfall-2, as well as the positive main effect for age at footfall-5 and footfall-6 showed that, increased age generally led to people taking longer steps early on in the walking task, but shorter steps (negative adjustments) as they drew closer to their target (i.e. the curb-like platform).

## Discussion

The aim of the current study was to describe age-related differences in locomotor pointing behavior in terms of when younger and older participants initiated the adjusting of their steps, what strategy they used when regulating their steps (lengthening, shortening or a mixed strategy) and the strength of perceptual-motor coupling in the approach when stepping up a curb. The main results relating to these aims are summarized in Table 2. The main findings of this study were that the variability in foot placement was lower for older adults and that younger and older adults used different step length adaptation strategies. Specifically, it was shown that younger participants preferred to lengthen their steps when making an adjustment, whereas older participants more often chose a shortening strategy in regulating to accurately place a footfall on top of the curb. Furthermore, confirming our hypothesis that older age would be associated with stronger perceptual-motor coupling, as participants drew nearer to the curb the relationship between the adjustment required in any single footfall and the adjustment produced in the following step (indicative of the strength of perceptual-motor coupling) became stronger with each additional step toward the curb, and with increasing age. No age-related differences were found in the onset of regulation, though it was found that regulation was initiated later when a lengthening strategy was chosen.

**Table 2:** Summary of main results relating to the aging process.

	Analysis type—Dependent variable	Main results relating to the effects of age
Onset of regulation	LME—OnsetReg	No effect of age was found
Perceptual-motor coupling	LME—Footfall SD / Step Length	Significant fixed effect for age indicated that older age was associated with lower scaled variability;
		Significant random effect of age at footfalls 0, -1, -4, -5 and -6 indicated that the influence of ageing was most pronounced at the end of the approach (i.e. closer to the target)
	LME—relationship between Adjust_required_ and Adjust_produced_	Significant fixed interaction effect for age and Adjust_required_ indicated that older age was associated with a stronger relation between Adjust_required_ and Adjust_produced_;
		Significant random effects at footfalls -1, -2, -5 and -6 indicated that the effect of age on the relation between Adjust_required_ and Adjust_produced_ was strengthened as participants got closer to the curb
Adjustment strategy	ANOVA—Number of trials using a strategy	Young group showed more lengthening walks, older groups showed more shortening trials; no differences between older groups.
	LME—Step Length	Significant fixed effect for age indicated older age was associated with taking shorter steps;
		significant random effect at step0 showed that the final step became especially short with increased age
	LME—relationship between Adjust_required_ and Adjust_produced_	Significant random effect of age at footfalls -1, -2, -5 and -6 indicated that participants took shorter steps towards the end of the walk if they were older

The strength of perceptual-motor coupling was studied as the relationship between the adjustment required and adjustment produced. A steeper regression line (or larger positive coefficients in LME modelling) for this relationship can be interpreted as representing a stronger perceptual-motor coupling. With increasing age, there was a stronger coupling between the perception of the amount of adjustment required in a single footfall and the adjustment produced in the next step, showing that perceptual-motor coupling was influenced by age (i.e. older age was related to stronger coupling).

It is worthwhile to consider whether the age-related differences in perceptual-motor coupling found can be linked to the decreasing action capabilities in the older cohort. It has been well-documented that ageing process influences the individual’s action capabilities [17–19]. For instance, reduced functioning in older adults is related to sarcopenia, the age-related loss of skeletal muscle mass [17], and it has been shown that older adults experience a reduced joint range of motion and decreased strength, which has been shown to influence gait stability [18]. As a result, it could be argued, that this allows them less adaptability in the regulation of their walking approach. In contrast, the action capabilities of the younger people, might allow them more adaptability in their movements. More movement adaptability might be related to a greater number of potential actions to successfully perform a movement task. For instance, in the task of crossing the road to step up a curb, the younger adults could choose between shortening and lengthening their strides. Therefore, the younger adults in the current study were less constrained by their action capabilities, which might have expressed itself in lower task demands, and subsequently, less strong perceptual-motor coupling in the final strides before stepping up the curb.

The effects of age on the strength of perceptual-motor coupling are relevant in relationship to the findings of previous studies. When comparing their findings from locomotor pointing experiments involving walking with findings of pointing in the long jump approach, Cornus and colleagues [8] argued that the later initiation of regulation in their participants was related to the lower spatiotemporal demands of walking compared to a running long jump approach [8]. This follows the reasoning that the strength of perceptual-motor coupling is varied relative to the spatiotemporal demands of the task. Consistent with this idea, participants are expected to exert stronger control when their action system’s tolerance is at risk of being exceeded; that is, when they need to operate near their action-boundaries. Conversely, when participants can operate well within the action system’s tolerance, and well within their action boundaries, less stringent control, and weaker (or more intermittent) perceptual-motor coupling will be observed [20]. In relation to the findings of the current study, this would suggest that age-related declines in motor function, and the related reduced adaptability, is associated with the finding that older participants showed stronger perceptual-motor coupling in the final step onto the curb. This finding could lead to new hypotheses for future studies into whether and how the system’s tolerance is perceived by older adults with possible implications for healthy aging interventions.

Another main finding was that the younger participants preferred to lengthen their steps when approaching the curb, whereas older adults generally chose a shortening strategy. This is in accordance with previous studies that have reported similar behaviors for younger and older adults when approaching a step or obstacle [21–23]. It has been argued that younger people aim to maintain forward momentum, both for the sake of progression as well as stability [20]. In contrast, older adults are more likely to slow down due to anxiety and to allow for more time to plan their movements [21]. In addition to these potential reasons, we propose an alternative explanation for this finding. As research has shown that older adults are less well attuned to their action capabilities [24–26], it is reasonable to argue that older adults are also less attuned to their maximal step length (i.e. their action boundary). If an older adult is uncertain of his or her action boundary, there may be greater uncertainty about whether lengthening their step would result in an action that was outside the safe area of their action capabilities. With this uncertainty, a shortening strategy might be perceived as the safer option. This explanation would fit well in the growing body of research that relates an inability to perceive one’s own capabilities to movement errors [27–29] and in particular falls in older adults [16,26,30,31].

The participants for the cohort of older adults were recruited from a local community of healthy older individuals. As such, these older adults may have had a greater interest in maintaining an active lifestyle. It is well described that people become more sedentary as they get older [32] and that many age-related declines can be slowed or reversed with regular exercise [33,34]. Given the significant variation that exists in the activity profiles of older adults, the ageing process must be considered a very individual process. That is, two people of the same chronological age may have very different activity levels and, hence, very different perceptual-motor function. This would call for a more functional approach to ageing research. That is, future research might seek to not merely outline the effects of age, but to focus more on functional variables that are more descriptive of the ageing process. Future studies should investigate whether the interpersonal differences in perceptual-motor coupling in locomotor pointing could be better explained using a functional variable (e.g. step length or perceptual-motor coupling) rather than chronological age [35].

### Conclusion

Perceptual-motor coupling was studied in an ageing cohort using a task that involved approaching and stepping onto a curb-like platform. Results showed that older age is associated with steeper positive regression slopes between the adjustment required in foot placement and the adjustment produced in the following step. In the context of this and similar research, this increase in regression slope can be interpreted as a stronger perceptual-motor coupling. This effect was particularly strong in the final steps before the step-up. An argument is put forward in which the decreasing action capabilities of the ageing cohort lead to an increase of task demands in stepping onto a curb, which could explain the stronger coupling shown by the older participants. Furthermore, it was found that younger adults, on average, lengthened their steps when regulating their step lengths during their approach toward a curb. Older adults did not show this increase in step length in the final steps and more often showed a shortening strategy. Future research should focus on the question whether this change in regulation is similar for all populations or the possibility that fall risk in older adults might be associated with differences in perceptual-motor coupling.

## Supporting information

S1 Table Fixed and random factors in a linear mixed effects model predicting normalized variability of foot placement (SD-footfall/Step Length). (DOCX)

S2 Table Fixed and random factors in a linear mixed effects model predicting the onset of regulation (OnsetReg). (DOCX)

S3 Table Fixed and random factors in a linear mixed effects model predicting Step Length (SL). (DOCX)

S4 Table Fixed and Random factors in a Linear Mixed Effects Model Predicting the Step Length adjustment produced in each step (Adjust_produced_). (DOCX)
