# Supplementary material for: Regulation of locomotor pointing across the lifespan: Investigating age-related influences on perceptual-motor coupling
Source: PLoS One. 2018 Jul 19;13(7):e0200244. doi: 10.1371/journal.pone.0200244 (PMC6053146; doi:10.1371/journal.pone.0200244)
Supplement: S1 Table — (DOCX) [file pone.0200244.s001.docx]

|  | Fixed Factors | | |
| --- | --- | --- | --- |
|  | Beta | SE | p value |
| Intercept | 0.278 | 0.025 | **< 0.001** |
| Age | >-0.001 | < 0.001 | **0.045** |
|  |  | | |
|  | Random Factors | | |
|  | Beta | Pred. SE | p value |
| Footfall 0 - Intercept | -0.109 | 0.0238 | **< 0.001** |
| Footfall 0 - Age | >-0.001 | < 0.001 | **< 0.001** |
| Footfall-1 - Intercept | -0.072 | 0.0238 | **0.003** |
| Footfall-1 - Age | >-0.001 | < 0.001 | **0.003** |
| Footfall-2 - Intercept | -0.012 | 0.0238 | 0.615 |
| Footfall-2 - Age | >-0.001 | < 0.001 | 0.615 |
| Footfall-3 - Intercept | 0.028 | 0.0238 | 0.241 |
| Footfall-3 - Age | < 0.001 | < 0.001 | 0.241 |
| Footfall-4 - Intercept | 0.050 | 0.0238 | **0.038** |
| Footfall-4 - Age | < 0.001 | < 0.001 | **0.038** |
| Footfall-5 - Intercept | 0.055 | 0.0238 | **0.021** |
| Footfall-5 - Age | < 0.001 | < 0.001 | **0.021** |
| Footfall-6 - Intercept | 0.059 | 0.0238 | **0.012** |
| Footfall-6 - Age | < 0.001 | < 0.001 | **0.012** |
| *Note*. P-values significant at an alpha of 0.05 are presented boldfaced | | | |
