# Supplementary material for: Regulation of locomotor pointing across the lifespan: Investigating age-related influences on perceptual-motor coupling
Source: PLoS One. 2018 Jul 19;13(7):e0200244. doi: 10.1371/journal.pone.0200244 (PMC6053146; doi:10.1371/journal.pone.0200244)
Supplement: S2 Table — (DOCX) [file pone.0200244.s002.docx]

|  | Fixed Factors | | |
| --- | --- | --- | --- |
|  | Beta | SE | p value |
| Intercept | -2.551 | 0.474 | **< 0.001** |
| Total Adjustment | -0.021 | 0.034 | 0.53 |
| Age | 0.004 | 0.006 | 0.495 |
| Total Adjustment*Age | < 0.001 | < 0.001 | 0.599 |
|  |  | | |
|  | Random Factors | | |
|  | Beta | Pred. SE | p value |
| Shortening - Intercept | -0.496 | 0.485 | 0.306 |
| Shortening - Total Adjustment | 0.019 | 0.034 | 0.589 |
| Shortening - Age | 0.012 | 0.006 | 0.062 |
| Shortening - Total Adjustment*Age | 0 | 0 | 0.374 |
| Lengthening - Intercept | 1.117 | 0.48 | **0.02** |
| Lengthening - Total Adjustment | -0.028 | 0.034 | 0.403 |
| Lengthening - Age | -0.011 | 0.006 | 0.085 |
| Lengthening - Total Adjustment*Age | 0 | 0 | 0.449 |
| Mixed - Intercept | -0.621 | 0.502 | 0.216 |
| Mixed - Total Adjustment | 0.01 | 0.035 | 0.782 |
| Mixed - Age | -0.001 | 0.007 | 0.878 |
| Mixed - Total Adjustment*Age | 0 | 0 | 0.889 |
| *Note*. P-values significant at an alpha of 0.05 are presented boldfaced | | | |
