# Supplementary material for: Regulation of locomotor pointing across the lifespan: Investigating age-related influences on perceptual-motor coupling
Source: PLoS One. 2018 Jul 19;13(7):e0200244. doi: 10.1371/journal.pone.0200244 (PMC6053146; doi:10.1371/journal.pone.0200244)
Supplement: S3 Table — (DOCX) [file pone.0200244.s003.docx]

|  | Fixed Factors | | |
| --- | --- | --- | --- |
|  | Beta | SE | p value |
| Intercept | 80.863 | 1.299 | **< 0.001** |
| Age | -0.19 | 0.021 | **< 0.001** |
|  |  | | |
|  | Random Factors | | |
|  | Beta | Pred. SE | p value |
| Step 0 - Intercept | 2.789 | 1.37 | **0.042** |
| Step 0 - Age | -0.054 | 0.027 | **0.042** |
| Step -1 - Intercept | -0.744 | 1.368 | 0.586 |
| Step -1 - Age | 0.014 | 0.026 | 0.586 |
| Step -2 - Intercept | 0.198 | 1.368 | 0.885 |
| Step -2 - Age | -0.004 | 0.026 | 0.885 |
| Step -3 - Intercept | 0.323 | 1.368 | 0.813 |
| Step -3 - Age | -0.006 | 0.026 | 0.813 |
| Step -4 - Intercept | -0.545 | 1.368 | 0.691 |
| Step -4 - Age | 0.011 | 0.026 | 0.691 |
| Step -5 - Intercept | -0.832 | 1.368 | 0.543 |
| Step -5 - Age | 0.016 | 0.026 | 0.543 |
| Step -6 - Intercept | -1.189 | 1.368 | 0.385 |
| Step -6 - Age | 0.023 | 0.026 | 0.385 |
| *Note*. P-values significant at an alpha of 0.05 are presented boldfaced | | | |
