# Supplementary material for: Regulation of locomotor pointing across the lifespan: Investigating age-related influences on perceptual-motor coupling
Source: PLoS One. 2018 Jul 19;13(7):e0200244. doi: 10.1371/journal.pone.0200244 (PMC6053146; doi:10.1371/journal.pone.0200244)
Supplement: S4 Table — (DOCX) [file pone.0200244.s004.docx]

|  | Fixed Factors | | |
| --- | --- | --- | --- |
|  | Beta | SE | p value |
| Intercept | < 0.001 | 0.119 | 0.997 |
| Adjust_required_ | 0.112 | 0.034 | **0.001** |
| Age | < 0.001 | 0.002 | 0.796 |
| Adjust_required_*Age | 0.001 | < 0.001 | **0.005** |
|  |  | | |
|  | Random Factors | | |
|  | Beta | Pred. SE | p value |
| Footfall-1 - Intercept | 0.203 | 0.064 | **0.002** |
| Footfall-1 - Adjust_required_ | 0.117 | 0.034 | **0.001** |
| Footfall-1 - Age | -0.003 | 0.001 | **0.001** |
| Footfall-1 - Adjust_required_*Age | 0.001 | 0 | **0.002** |
| Footfall-2 - Intercept | 0.146 | 0.063 | **0.022** |
| Footfall-2- Adjust_required_ | 0.096 | 0.034 | **0.005** |
| Footfall-2 - Age | -0.002 | 0.001 | **0.015** |
| Footfall-2 - Adjust_required_*Age | 0.001 | 0 | **0.004** |
| Footfall-3- Intercept | 0.001 | 0.063 | 0.99 |
| Footfall-3 - Adjust_required_ | 0.01 | 0.034 | 0.762 |
| Footfall-3 - Age | 0 | 0.001 | 0.937 |
| Footfall-3 - Adjust_required_*Age | 0 | 0 | 0.568 |
| Footfall-4 - Intercept | -0.06 | 0.063 | 0.341 |
| Footfall-4 - Adjust_required_ | -0.045 | 0.034 | 0.194 |
| Footfall-4 - Age | 0.001 | 0.001 | 0.297 |
| Footfall-4 - Adjust_required_*Age | 0 | 0 | 0.138 |
| Footfall-5 - Intercept | -0.116 | 0.063 | 0.064 |
| Footfall-5 - Adjust_required_ | -0.077 | 0.034 | **0.025** |
| Footfall-5 - Age | 0.002 | 0.001 | **0.05** |
| Footfall-5 - Adjust_required_*Age | -0.001 | 0 | **0.02** |
| Footfall-6 - Intercept | -0.173 | 0.063 | **0.006** |
| Footfall-6 - Adjust_required_ | -0.102 | 0.034 | **0.003** |
| Footfall-6 - Age | 0.003 | 0.001 | **0.005** |
| Footfall-6 - Adjust_required_*Age | -0.001 | 0 | **0.006** |
| *Note.* P-values significant at an alpha of 0.05 are presented boldfaced | | | |
